# Signaling the trustworthiness of science

**DOI:** 10.1073/pnas.1913039116

**Published:** 2019-09-23

**Authors:** Kathleen Hall Jamieson, Marcia McNutt, Veronique Kiermer, Richard Sever

**Affiliations:** ^a^Annenberg School for Communication, University of Pennsylvania, Philadelphia, PA 19104;; ^b^National Academy of Sciences, Washington, DC 20001;; ^c^Public Library of Science, San Francisco, CA 94111;; ^d^Cold Spring Harbor Laboratory, Cold Spring Harbor, NY 11724

**Keywords:** scientific integrity, transparency, signaling trustworthiness

## Abstract

Trust in science increases when scientists and the outlets certifying their work honor science’s norms. Scientists often fail to signal to other scientists and, perhaps more importantly, the public that these norms are being upheld. They could do so as they generate, certify, and react to each other’s findings: for example, by promoting the use and value of evidence, transparent reporting, self-correction, replication, a culture of critique, and controls for bias. A number of approaches for authors and journals would lead to more effective signals of trustworthiness at the article level. These include article badging, checklists, a more extensive withdrawal ontology, identity verification, better forward linking, and greater transparency.

Although confidence in science remains high (1), recent decades have seen instances in which individuals, research institutes, and scholarly outlets have failed to embody the competence, integrity, and benevolence required to sustain perceptions that science is trustworthy. Some concerns have been generated by instances of misconduct or fraud (2, 3); others have arisen as a consequence of failures to replicate key findings (4, 5) and a rise in the number of retractions (6). In response, the research community has started institutionalizing practices designed to thwart human biases and increase the trustworthiness of scholarly work. Yet, there has been no corresponding community agreement on optimal ways to signal the existence and application of such practices within the outputs and narratives generated.

This absence is problematic. Without clear signals, other scientists have difficulties ascertaining confidence in the work, and the press, policy makers, and the public at large may base trust decisions on inappropriate grounds, such as deeply held and irrational biases, nonscientific beliefs, and misdirection by conflicted stakeholders or malicious actors.

## Signaling Trustworthiness of the Scientific Enterprise to the Public

**Fig. 1.**
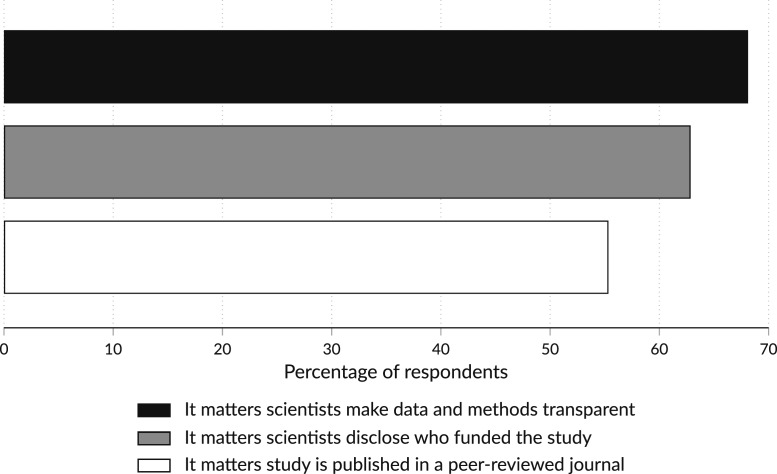
A national probability sample of 1,253 US adults conducted for the Annenberg Public Policy Center of the University of Pennsylvania via telephone by Social Science Research Solutions from January 30 to February 7, 2019. The margin of error is ±3.42% at the 95% confidence level. Total cellular phone respondents were 854 (68% of the sample) while 399 respondents (32%) completed the survey using a landline. There were 39 respondents (3%) who completed the survey in Spanish. The response rate, which was calculated using the American Association for Public Opinion Research’s Response Rate 3 formula, was 7%. See the *SI Appendix* and Dataset S1 for additional details.

Science is trustworthy in part because it honors its norms. Adherence to these norms increases the reliability of the resulting knowledge and the likelihood that the public views science as reliable. A 2019 survey (Fig. 1) found that the public recognizes key signals of the trustworthiness of scientific findings. Specifically, in deciding whether to believe a scientific finding, 68% said it matters whether the scientists make their data and methods available and are completely transparent about their methods; 63% reported that it matters whether the scientists involved in the study disclose the individuals and organizations that funded their work; and 55% indicated that it matters whether the study has been published in a peer-reviewed science journal.

Given this critical recognition and the need to sustain it in a changing communication landscape, we propose a variety of mechanisms that would better communicate the practices that embody scientific norms both at the system level—that is, how scientists communicate, certify, and react to findings— and at the level of individual studies and study reports (articles).

## Communicating Practices That Reinforce the Norms

Practices that scientists use to reinforce the norms of science may not be immediately transparent to a more general audience. Moreover, they can be misunderstood, or the message may be manipulated by other interests to create misinformation. Researchers can improve the understanding of how the norms of science are honored by communicating the value of these practices more explicitly and transparently and not inadvertently supporting misconceptions of science.

Although science advances as researchers build on each other’s work, it does not progress in a straight line, but rather in fits and starts, with vast leaps and technological revolutions opening new areas of inquiry and sometimes, in the process, correcting previous interpretations. Yet researchers sometimes write their papers as though the result had been anticipated all along, reinforcing the public perception that scientists can engineer whatever findings they desire. Central to the progress of science is a culture of critique, replication, and independent validation of results, and self-correction. The Mertonian norm of communalism (7), by which scientists share their results and methods transparently to promote collaboration and organized skepticism, is essential to these practices.

### Communicating That Science Champions a Culture of Critique.

Science’s culture of critique discourages groupthink, countermands the effects of human biases, and protects knowledge, not only by rewarding a dispassionate stance toward the subject and institutionalizing organized skepticism but also by fostering competition among scientists able to replicate and hence challenge each other’s work. However, to those outside the research community, this attitude and set of practices can make scientists seem detached, unfeeling, and/or contrarian, a perception that can undermine trust that scientists are acting with goodwill.

Moreover, disagreement among scientists who favor alternative hypotheses can invite doubts among those in the public who mistakenly view science as a collection of facts cascading toward incontrovertible knowledge in a linear fashion. Scholars inadvertently reinforce this viewpoint by only rarely explaining how new evidence has led them to update or reject an earlier result. One of the more effective ways to assure the public of the integrity of science is featuring instances in which scientists recount the process and evidence that led them to reconsider a previously held view (8).

### Communicating the Value Placed on Replication and Transparency.

Other signals of trustworthiness indicate the extent to which the research community encourages transparency. As a prerequisite to scrutiny of studies, transparency supports trust by allowing others to examine study design, execution, and data and, as a result, replicate results. Archiving data and analysis plans in publicly available repositories makes it possible to both validate and build upon the results of others. Preregistration of analysis plans establishes that the authors have intended from the start to test a stated hypothesis and helps minimize investigator and publication biases.

Before transparency can become commonplace, however, other community actions are required, such as development of additional infrastructure for data archiving and procedural standards to maintain ethics, in particular where consent and privacy are relevant. The scientific community also needs to propagate standards that will facilitate data discovery and reuse, such as the Findability, Accessibility, Interoperability, and Reusability (FAIR) framework (9).

### Signaling Adherence to the Norm of Self-Correction.

A powerful driver of reliable knowledge is science’s incentivizing of self-correction. As a way of knowing, science has never claimed infallibility. Clear and accountable retractions (and, as proposed below, broader categories of corrections to the record, such as “voluntary withdrawals”) are central elements in the advancement of science—likewise corrigenda and errata.

Part of that accountability relies on institutions (universities, journals, and funders) establishing robust procedures to investigate and/or communicate the results when findings are suspect, whether as a result of honest error or fraud. Accordingly, journals should use forward and back links to tie “editorial expressions of concern,” retractions, and other updates to suspect articles.

The relevant community should also ensure that indexing services (e.g., PubMed, Google Scholar, and DOI-registration agencies) and downstream elements, such as citations in derivative work, are updated. Tools such as Crossref Event Data (10) and Crossmark (11) provide mechanisms to serve this end and should be widely adopted.

Just as attaching a retraction notice to a study’s metadata signals that work’s unreliability, adding links to replications of a published finding would signal additional evidence of trustworthiness. Such addenda, which would not have been possible in the print era, can be more easily implemented in the digital age. Whenever practical, enabling “forward linking,” not just backward citation, to essential articles can establish a chain of trust. Of course, viewed across time, scientific findings are subject to change. Neither alterations in interpretation that are part of the normal progress of science nor findings eclipsed by advances in knowledge warrant retractions.

Better incentives and procedures are also needed to maximize the likelihood that universities carry out and complete investigations of flawed research by scholars in their employ.

### Communicating the Ways in Which Science Certifies the Integrity of Its Evidence and Inferences.

The research community should clearly signal its reliance on safeguards that require scholars to test the integrity of their evidence and the legitimacy of the inferences they have drawn from it.

Journals play a key role in ascertaining the quality of the evidence in work submitted for publication through peer review. However, that practice is neither uniform nor free of exploitation. While it can select for trustable results, its existence alone is not sufficient to verify the quality of the evidence used to back claims. Unfortunately, there is no independent body that certifies the process or quality of peer review.

In the worst scenario, some disreputable journals claim peer review without performing it (12). Knowingly or unknowingly, authors publish their work through these outlets and obtain credit with promotion committees for scholarly contributions that may not have had any true scrutiny or evaluation. But even reputable journals differ from each other in their peer review standards, and biases have been identified across the literature (13–16).

Additionally, the anonymous character of most peer review has led to cases of fraud in which authors or their agents have posed as independent reviewers (e.g., peer review rings) (17). One development with the potential to thwart reviewer fraud is the use of Open Researcher and Contributor iDs (ORCID iDs) (https://orcid.org/), unique persistent digital identifiers for researchers. ORCID iDs can help disambiguate individuals and have the potential to establish the authenticity of their credentials by linking with their affiliations and record of published works.

Importantly, because peer review is, for the most part, conducted confidentially, its role in protecting the integrity of science is not transparent to readers. An increasing number of journals are making the peer review process more open by publishing reviewer reports (18) (without attribution necessarily). This practice should facilitate both assessments of the effectiveness of peer review and the establishment of concrete measures of quality (19).

### Adopting Signaling Language That Is Clear and Appropriately Aligns with Productive Incentives.

The scientific community has recognized that precise specification is a scientific norm and that community agreement on nomenclature is a key component of effective signaling (20–22). Responding to calls for standardized definitions of such key concepts as reproducibility and replicability (20), a 2019 National Academies of Sciences, Engineering, and Medicine (NASEM) report (21) provided much-needed clarity. Yet the language in which scientists communicate a number of central activities remains imprecise and, in some instances, counterproductive. For example, the language in which decertification of problematic findings is conveyed is inexact. A single term, “retraction,” is used to refer both to a voluntary withdrawal of a paper from a journal by the authors following the discovery of an unintended error and to the involuntary removal of a paper from a journal following an investigation that uncovers scientific misconduct by 1 or more of the authors. The use of the same term in these dissimilar cases may discourage honest authors from coming forward to amend the literature. Leading the way in implementing change are preprint servers, such as bioRxiv and arXiv, that use the term “withdrawal” rather than “retraction” to denote papers whose authors wish them no longer to be considered part of the scientific record. Alberts et al. (23) have suggested “voluntary withdrawal” for the former and “withdrawal for cause” for the latter to distinguish the 2 cases. Other circumstances may lead to “editorial withdrawal.” Without recommending specific language, we wish to see more nuance in the standard terms used to correct the scientific record.

Most journal editors publish statements of retraction that identify the issue that led to the decertification (e.g., whether the data or analyses were flawed) to the extent that it is known and, when possible, who was responsible for the paper’s shortcomings. In cases in which an official investigation has established misconduct by a subset of the authors, this practice avoids placing blame more broadly than warranted.

A second counterproductive usage is “conflict of interest” to encompass relationships that should be disclosed. This commonplace phrase implies that all such ties necessarily corrupt the research, which is not the case. Use of a more neutral term would encourage vigilance without disincentivizing disclosure. “Competing interest” is used by many journals. “Relevant interest” or “relevant relationship” might be more appropriate.

## Signaling Norms at the Study Level

Researchers can better communicate that they have adhered to the norms of their field when communicating the results of individual studies in scholarly works (Table 1). Development of standards will necessarily reside in the fields, for example, through the efforts of disciplinary societies. Journals can promote this adherence by requiring standards for reporting studies (24) and verification, thus more effectively conveying whether a study is trustworthy.

**Table 1.** Signaling the trustworthiness of studies

Dimension	Norms	Example of violation	Signaling trust at the study level	Role of stakeholders
Competence	Bias-minimizing and power-optimizing study design	Insufficient power, selective sampling, absence of bias- controlling measures (e.g., blinding, randomization)	Signal that study meets standards of reporting transparency	Researchers increase trust by reporting on ways they meet norms and sharing details of methods, code, and data—by providing links with PIDs.
	Reliance on statistics	p-hacking (29)	Signal that statistical review has been conducted	
	Use of reliable reagents	Use of invalidated biological reagents	Report on reagent validation	Journals and publishing platforms develop and enforce reporting standards and make clear what has been verified in review. As such they become trusted vehicles.
	Distinction between exploratory and confirmatory studies	HARKing (30), use of inferential statistics in exploratory studies, outcome switching	Preregistration of hypothesis-testing studies; clarity about post hoc analyses	
	Conclusions supported by data	Hyped results, data not available	Modest reporting, analysis transparency, data available (with persistent identifier)	Research institutions provide education, environment, and infrastructure to support best practices (e.g., testing reagents, managing data, incentivizing rigor in tenure and promotion).
Integrity	Transparency of competing interests	Hidden interests with potential to influence study outcome	Disclosure of competing interests	Journals ensure independence of peer reviewers, use ORCID to attribute activities to individuals, and use available technology for checks.
	Validation by peer review	Failure to open data, methods, materials to scrutiny	Open data and materials policies	
		Subversion of peer review (e.g., reviewer rings)	Publish/verify identity of reviewers	
	Ethical treatment of research subjects and animals	Inadequate or nonexistent informed consent	Report on IRB approval and permits obtained	Research institutions provide infrastructure such as IRB with relevant expertise.
		Failure to obtain research permits		Research institutions and funders make research ethics a condition of support, provide education, and facilitate access to permit- granting organizations.
Benevolence	Disinterestedness	Financial, personal, or political interests leading to false or selective reporting	Expressions of concern and retractions issued when misconduct is demonstrated or results do not support conclusions	Authors increase trust by being transparent about potential competing interests.
				Research institutions investigate misconduct fairly and rapidly, report the outcomes, and protect whistleblowers.
				Journals collaborate with institutions to investigate allegations of data falsification and act to protect the record.
				Overarching structures (e.g., National Academies) establish norms and arbitration mechanisms (e.g., ombuds).

The table presents a variety of mechanisms that can help communicate the level of trust warranted by an individual study. Although, in isolation, none is a fail-safe, collectively signaling their presence should help certify the trustworthiness of a study, researcher, or field of inquiry. HARKing (hypothesizing after the results are known) presents a post hoc hypothesis as if it were an a priori one (30); p-hacking is a form of data dredging that occurs “when researchers try out several statistical analyses and/or data eligibility specifications and then selectively report those that produce significant results” (29); IRB, institutional review board; PIDs, persistent identifiers.

### The Characteristics of a Strong Signal.

The capacity of a signal to communicate trustworthiness is enhanced if it has the following characteristics: 1) The message it sends is unambiguous and consistently conveyed (“trust this research” or “trust that this research properly followed a specific norm”); 2) the signal is unavoidable and its referent obvious (the quality of the data underlying a figure, the reproducibility of results in a paper, or the integrity of peer review of a journal); 3) the signal cannot be readily counterfeited (for example, it supplies provenance and links to forms of certification readily verifiable by someone other than the signaler); 4) the signal is delivered by a trusted, trustworthy, unbiased, and unconflicted source; and 5) the signal is designed with the receiver in mind (e.g., multiple, nuanced signals for researchers; simpler, high-level signals for nonresearchers).

### Vehicles That Signal Trustworthiness.

Checklists and badges are among the means authors, journals, and publishing platforms can use to signal the trustworthiness of a study or a finding.

### Checklists.

By adopting a checklist describing its expectations of manuscripts, a journal or publishing platform can communicate the ways in which it protects the evidence-gathering and -reporting process. These expectations should include information on each author’s contributions to the study, relevant potentially biasing relationships, and how to access data, materials, and computer code; they should also indicate reporting requirements about materials, experimental and analytical design, and statistical tests and confirm adherence to field-specific reporting requirements. Some journals (25, 26) already make use of these checklists with variable results. Refinement and standardization are desirable (27), for authors as well as readers.

To verify the evidentiary basis for the research they publish, many journals now complement peer review with checks to detect plagiarism and image manipulation, independent statistics checks, and verification that the authors have complied with community-endorsed reporting and archiving standards. Signaling the extent to which a journal employs these forms of verification could also increase trustworthiness.

### Badges.

**Fig. 2.**
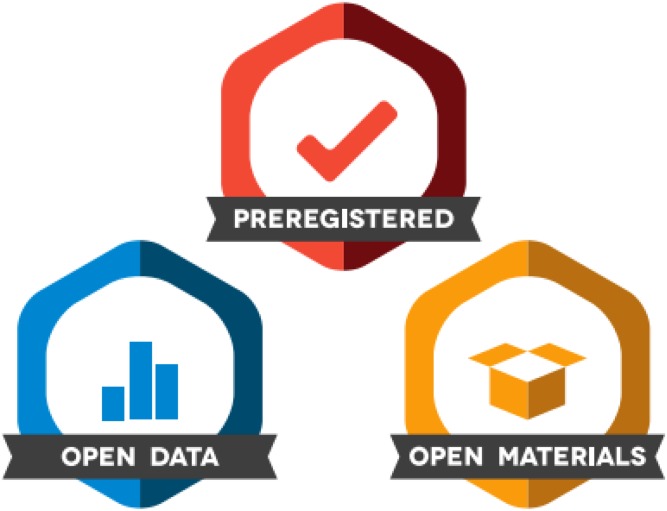
Examples of badges offered by the Center for Open Science that can be adopted by journals. Badges recognize those studies that meet standards for open data, open materials, and preregistration (https://cos.io/).

Currently the Center for Open Science (https://cos.io/) signals elements of trustworthiness (such as openness) by offering badges (Fig. 2), not only for studies that comply with guidelines for open data and open materials but also for those that were preregistered and followed the protocol specified in the preregistration. As journals have adopted such badging, their editors have observed that author compliance with journal policies has increased (28) although other factors may have contributed as well.

## Conclusion

Science enjoys a relatively high level of public trust. To sustain this valued commodity, in our increasingly polarized age, scientists and the custodians of science would do well to signal to other researchers and to the public and policy makers the ways in which they are safeguarding science’s norms and improving the practices that protect its integrity as a way of knowing.

Embedding signals of trust in the reporting of individual studies can help researchers build their peers’ confidence in their work. Publishing platforms that rigorously apply these signals of trust can increase their standing as trustworthy vehicles. But beyond this peer-to-peer communication, the research community and its institutions also can signal to the public and policy makers that the scientific community itself actively protects the trustworthiness of its work.

## Supplementary Material

Supplementary File

Supplementary File
