# Supplementary material for: Signaling the trustworthiness of science
Source: Proc Natl Acad Sci U S A. 2019 Sep 23;116(39):19231–6. doi: 10.1073/pnas.1913039116 (PMC6765233; doi:10.1073/pnas.1913039116)
Supplement: Supplementary File [file pnas.1913039116.sapp.pdf]

SI APPENDIX

Q2. I'm going to read you a list of items. For each one, please tell me how much it matters to you when you are deciding whether to believe a scientific finding. Please use a scale from 1 to 5, where 1 means it "Does NOT matter at all" and 5 means it "Matters a great deal". Of course, you may use any number in between.
Here's the first: (READ FIRST ITEM). How much does that matter to you when deciding whether to believe a scientific finding? Please use a scale from 1 to 5, where 1 means it "Does NOT matter at all" and 5 means it "Matters a great deal". Of course, you may use any number in between. How about (INSERT NEXT ITEM)?

a. The study has been published in a peer-reviewed science journal

	**1 – Does not matter at all (%)**	**2 (%)**	**3 (%)**	**4 (%)**	**5 – Matters a great deal (%)**	**Don't know (%)**	**Refused (%)**
2019	9.0	9.8	19.1	24.1	37.4	0.6	-

b. The scientists make their data and methods available and are completely transparent about their methods

	**1 – Does not matter at all (%)**	**2 (%)**	**3 (%)**	**4 (%)**	**5 – Matters a great deal (%)**	**Don't know (%)**	**Refused (%)**
2019	7.2	7.5	12.8	16.4	55.7	*	*

*=Less than 0.5%

e. The scientists involved in the study disclose the individuals and organizations that funded their work

	**1 – Does not matter at all (%)**	**2 (%)**	**3 (%)**	**4 (%)**	**5 – Matters a great deal (%)**	**Don't know (%)**	**Refused (%)**
2019	8.4	9.2	15.4	15.9	50.7	*	-

*=Less than 0.5%

www.pnas.org/cgi/doi/10.1073/pnas.1913039116

Methodology

A national probability sample of 1,253 US adults conducted for the Annenberg Public Policy Center of the University of Pennsylvania via telephone by Social Science Research Solutions from January 30 to February 7, 2019. The margin of error is +/- 3.42% at the 95% confidence level. Total cellular phone respondents were 854 (68% of the sample) while 399 respondents (32%) completed the survey using a landline. There were 39 respondents (3%) who completed the survey in Spanish. Data were weighted to represent the target U.S. adult population. The response rate, which was calculated using the American Association for Public Opinion Research's Response Rate 3 formula, was 7%.
